# Supplementary material for: Food Consumption of People with Sickle Cell Anemia in a Middle-Income Country
Source: Nutrients. 2023 Mar 19;15(6):1478. doi: 10.3390/nu15061478 (PMC10054003; doi:10.3390/nu15061478)
Supplement: Supplementary file 1 [file nutrients-15-01478-s001.zip › nutrients-2208687-supplementary.pdf]

Table S1. Synthesis of the referred items in the 24-hour-recalls grouped according to the NOVA classification.

NOVA food groups	Referred items
In natura or minimally processed foods (including culinary preparations based on these foods)	avocado<br>pumpkin<br>zucchini<br>açaí<br>leafy vegetables<br>Coconut Water<br>cassava<br>lettuce<br>red plum<br>banana<br>potato<br>sweet potato<br>eggplant<br>beet<br>broccoli<br>khaki<br>onion<br>carrot<br>chayote<br>coconut<br>coriander<br>cabbage<br>cauliflower<br>guava<br>tubers<br>Scarlet eggplant<br>orange<br>lychee<br>lemon<br>apple<br>papaya<br>watermelon<br>melon<br>corn<br>palm heart<br>strawberry<br>orange juice<br>tangerine<br>tomato<br>grape<br>cucumber<br>pear<br>pepper<br>cabbage<br>fruit salad<br>okra

	cane juice<br>sugar<br>olive oil<br>butter<br>honey<br>White rice<br>rice with meats<br>rice with vegetables<br>brown rice<br>beans<br>oat<br>beef<br>meatball<br>shrimp<br>pork<br>chicken<br>liver and other viscera<br>omelet<br>egg<br>fish<br>soy kafta<br>nuts<br>flour<br>skimmed milk<br>whole milk<br>oatmeal flour<br>cassava flour<br>cake<br>bean broth<br>Chicken Soup<br>spinach cream<br>couscous<br>farofa<br>pasta<br>oatmeal porridge<br>pancake<br>chickpea paste<br>mashed potato<br>risotto<br>Chicken salad<br>soup<br>Sushi<br>tapioca<br>tea<br>coffee
Processed foods	French bread and other processed breads<br>processed cheeses<br>canned tuna<br>olive<br>canned green corn

	canned fruit<br>jam<br>dried meat<br>beer
Ultra-processed foods	sliced bread and other ultra-processed breads<br>sweets and candies<br>chocolate<br>gelatin<br>flavored yogurt<br>condensed milk<br>peanut candy<br>pudding<br>popsicle<br>ice cream<br>Dairy beverage<br>Gatorade<br>soft drinks<br>chocolate powder<br>juice powder<br>ultra-processed cake<br>stuffed sweet biscuit<br>sweet biscuit without filling<br>cracker<br>cornflakes<br>popcorn<br>baked salted<br>fried snacks<br>packet snacks<br>French fries<br>hot dog and other sandwiches<br>farofa ready<br>hamburger<br>frozen lasagna<br>noodles<br>Fried pastry<br>Pizza<br>ready soup<br>mortadella<br>nuggets<br>sausage<br>Ham<br>Pâté Ham<br>Turkey breast<br>ultra-processed cheeses<br>cream cheese<br>ketchup, mayonnaise and other prepared sauces<br>Margarine
